# Selections and Abstracts

**Published:** 1901-05

**Authors:** 


### SELECTIONS AND ABSTRACTS.

H. PLYMPTON, M.D., No. 2 Macon street, Brooklyn, N. Y., writes:

Just a brief history of two cases of endometritis treated by me with gratifying results, due to Glyco-Thymoline:

CASE NO. 1. Mrs. F——, December 30, endometritis extensive. Os patulous and inflamed, ulceration on os and floor of vagina; constant pain in lumbar region; severe nervous phenomena, profuse discharge. Had tried formaldehyd, zinc sulphate, nitrate of silver applications, pure carbolic, ichthyol, etc., without anything more than temporary relief. Condition had existed since early in November.

Glyco-Thymoline applied by means of tampon of cotton saturated; this application following a douche of water (110° F.) two quarts, Glyco-Thymoline about four ounces. Tampon applied twice a day and allowed to remain in position three hours. Relief experienced, especially from pain and nervous symptoms, in twenty-four hours. Improvement steady with no setbacks till January 10th, when tampons were omitted and douches (three per day) only continued.

January 29th. Examination revealed no inflammation, no discharge, entire relief from backache and nervous symptoms.

CASE NO. 2. Mrs. E——, same condition prevailed as in Case No. 1, only environment not so favorable. This patient not so able to care for herself as the one previously reported.

Took charge November 16th, and followed same treatment, viz.: Glyco-Thymoline by tampon and douche. By January 20th the inflammation had disappeared entirely and the patient was discharged. Had her surroundings been such as to make it possible for her to follow directions carefully, the recovery would have been earlier by two or three weeks.

